# Notice Respecting Dr. Schultzenheim

**Published:** 1814-10

**Authors:** John Christian Wachsel

**Affiliations:** Resident Apothecary, &c. &c. at the Hospitals for the Small-pox, for Inoculation and for Vaccination, at Pancras


*For the Medical and Physical Journal.*

*Notice respecting Dr. Schultzenheim; by* Mr. J. C. WACHSEL.

IN the Note on the Report of Vaccination in Sweden, page 250 in your last Number, Dr. Schultzenheim is described as appointed in the year 1754, by the States of the Kingdom, to inquire into Sutton's and Dimsdale's mode of inoculation in England. In justice to Dr. Archer and these Hospitals, it is, that I acquaint you, Dr. Schultzenheim was for twelve months a pupil of Dr. Archer's, and so diligent in the attendance on the practice of these Hospitals, that he afterwards published a very correct account of that institution. His work was printed for A. Linde, in Catherine-street in the Strand, in the year 1758.

JOHN CHRISTIAN WACHSEL,

*Resident Apothecary, &c. &c. at the Hospitals for the Small-pox, for Inoculation and for Vaccination, at Pancras.*

*Sept.* 14, 1814.

